# Relationship between ozone and temperature during the 2003 heat wave in France: consequences for health data analysis

**DOI:** 10.1186/1471-2458-6-261

**Published:** 2006-10-20

**Authors:** Sandrine A Lacour, Michèle de Monte, Patrice Diot, Jérôme Brocca, Nadège Veron, Patrice Colin, Valérie Leblond

**Affiliations:** 1 Inserm, U618, Tours, F-37000 France; Univ François Rabelais, Tours, F-37000 France; IFR 135, Tours, F-37000, France; 2 DRSM Centre, Orléans, F-45000, France; 3 Lig'Air, Orléans, F-45000, France

## Abstract

**Background:**

PAPRICA is a research program designed to estimate the impact on the health of patients with chronic respiratory insufficiency of a prevention strategy based on notification of ozone pollution. The first year of this study was conducted during the 2003 heat wave, and high temperatures were therefore considered as a confounding factor in the data analysis. The aim of the present study was to assess the relationship between ozone and temperature in order to propose a methodology to distinguish between the effects of ozone and temperature on the impact of a prevention strategy with regard to ozone pollution.

**Methods:**

Multivariate analyses were used to identify associated climate and ozone pollution profiles. This descriptive method is of great value to highlight the complexity of interactions between these parameters.

**Results:**

Ozone concentration and temperature were strongly correlated, but the health impact of ozone pollution alone will be evaluated by focusing on situations characterized by ozone concentrations above 110 μg/m^3^/8h (air quality guidelines to protect human health defined by the French legislation) and temperatures lower than 26°C, below the discomfort threshold.

**Conclusion:**

The precise relationship between ambient ozone concentration and temperature identified during the PAPRICA 2003 study period will be used in analysing the PAPRICA health data.

## Background

In Europe, the public must be informed when ozone concentrations reach a threshold of 180 μg/m^3^/h (the concept of threshold means in this context the value above which an information is given). This strategy of information is justified by many epidemiological studies which have reported that high levels of ozone in the air are associated with an increase in acute mortality [1-3] and morbidity [4-6].

Other studies suggest that ozone concentrations can have harmful effects on health at even lower levels when they last for several days [7-9]. In France, the objectives of air quality control are to maintain ozone concentrations less than 110 μg/m^3 ^averaged over 8 hours. In the Centre Region of France, these ozone levels are reached or exceeded on average 60 days per year between April and September, but the public does not receive specific information.

Within this framework, the network of public health research, PAPRICA, was created in 2002. This network brings together various partners of the Centre Region concerned with air pollution and its impact on health and engaged in research into the effect of ozone on health. Modelling software was used in the Centre Region to predict 24 hours in advance when ozone would exceed 110 μg/m^3 ^for 8 hours. A group of patients with severe chronic respiratory deficiency were informed the day before these levels were expected to be exceeded and were given written recommendations about how to protect themselves against ozone exposure. A matched group of patients did not receive this information. The impact of this information and prevention strategy on health was assessed through comparative analysis of the medical treatment of the two groups.

The first year of the PAPRICA study was conducted during the exceptionally hot summer of 2003, in which 14,800 deaths were recorded in France during the 9 days of extreme temperatures [10-12]. Consequently, the PAPRICA data analysis had to take into account the two parameters to which patients were exposed, i.e. high temperature and ozone.

The aim of the present study was to analyse the relationship between ozone and temperature in order to distinguish between the effects of high ozone concentration and high temperature on the impact of a prevention strategy with regard to ozone pollution.

## Methods

### Study area

The study was carried out between April 1 and September 30, 2003 in the cities of Chartres, Orléans and Tours in the Centre Region of France. Chartres is located in the north of the region 155 meters above sea level, Orléans in the north east 125 meters above sea level, and Tours in the south west 108 meters above sea level.

### Pollutant measurements

Air pollution data for ozone (O_3_) were provided by the regional air-quality network, Lig'Air [13]. Ozone data were recorded at urban and suburban monitoring stations: 2 urban stations in Chartres, 2 urban and 1 suburban stations in Orléans, and 3 urban and 1 suburban stations in Tours. Ozone concentrations were expressed in hourly values and in 8-hour moving averages. Medians of 1- and 8-hour ozone concentrations were determined for each city.

### Meteorological parameters

The meteorological parameters were temperature, relative humidity, atmospheric pressure, sunshine, and wind speed and direction, and were provided by the regional centre of Météo France [14].

### Statistical test

Pollutant measurements and meteorological parameters were analysed by Multiple Correspondence Analysis (MCA) using SPAD^® ^software as previously described [15].

MCA is a descriptive multivariate (multifactorial) method used to visualize a large number of variables on a same graph and to estimate the statistical relationship between variables. A descriptive multivariate analysis is particularly appropriate to assemble complex parameters with respect to multiple variables.

MCA was performed using the whole table of data with the 4,392 observations in rows (24 hours per day × 30 or 31 days per month × 6 months) and 8 variables in columns. These qualitative variables were sorted into categories which were noted in each column for each hour of a day of a month (statistical unit):

- Temperatures (T), expressed in degrees Celsius, were sorted into 7 categories: T1 = [- 4; 6[; T2 = [6; 11[; T3 = [11; 16[; T4 = [16; 21[; T5 = [21; 26[; T6 = [26; 31[; T7 = [31; 41[

- Relative Humidity (RH) values, expressed as a percentage of maximum moisture in the air, were sorted into 4 categories: RH1 = [0; 40[; RH2 = [40; 60[; RH3 = [60; 80[; RH4 = [80; 100[

- Sunshine (S), expressed in minutes of sunshine per hour, was sorted into 7 categories: S1 = 0; S2 = [1; 10]; S3 = [11; 20]; S4 = [21; 30]; S5 = [31; 40]; S6 = [41; 50]; S7 = [51; 60]

- Wind Directions were the different points of the compass card: North (N); North East (NE); East (E); South East (SE); South (S); South West (SW); West (W); North West (NW); plus Indetectable Wind (IW)

- Wind Speeds (WS), expressed in ms^-1^, were ranged from 0 to 13 ms^-1 ^and classified using the Beaufort scale: WS1 = Calm wind : 0 to 3.9 ms^-1^; WS2 = Moderate wind : 4 to 7.9 ms^-1^; WS3 = Rather strong wind : 8 to 13.9 ms^-1^

- Atmospheric pressures adjusted to sea level and expressed in hectopascal were sorted into 3 categories: P1 = Depression : [990; 1005[; P2 = Normal atmospheric pressure : [1005; 1020[; P3 = Anticyclone : [1020; 1035[

- 1- and 8-hour ozone concentrations, both expressed in μg/m^3^, were sorted into 6 categories: 1h-O_3_1 and 8h-O_3_1 = [0; 50[; 1h-O_3_2 and 8h-O_3_2 = [50; 80[; 1h-O_3_3 and 8h-O_3_3 = [80; 100[; 1h-O_3_4 and 8h-O_3_4 = [100; 120[; 1h-O_3_5 and 8h-O_3_5 = [120; 150[; 1h-O_3_6 and 8h-O_3_6 = [150; 260[.

The 6 active variables of the analysis were meteorological parameters (temperature, relative humidity, sunshine, atmospheric pressure, wind speed and direction) and the 2 illustrative variables were pollution levels (1- and 8-hour ozone concentrations).

MCA allows to construct a space in which 2 different categories of active variables are close together if they simultaneously appear several times in the whole table of data. For example, if temperatures comprise between 31 and 40°C (T7) are frequently associated with relative humidity values comprise between 0 and 40 % (RH1), these 2 categories will be close to each other in the space. The different categories of active variables are positioned in a multidimensional space, the number of dimensions of the space being proportional to the number of categories of actives variables. To observe the distance between categories on a 2-dimensional graph, the cloud of categories is projected on a plane of 2 orthogonal factors, F1 and F2. These factors were chosen as the most representative of the global variance of the cloud. In a second step, the supplementary (illustrative) variables are placed on the graph according to their proximity with the different categories of active variables.

We checked that each category of each variable was sufficiently represented inside the variable to apply the statistical tests, i.e. above √3n (n = 4392).

The relationship between meteorological parameters and pollutant measurements was evaluated using the DEMOD (DEscription of MODalities) procedure of SPAD which statistically characterizes each category of 1- and 8-hour ozone concentrations in relation to each climate parameter category. A chi square was calculated between the proportion of a given category of a given variable inside another category of another variable and the proportion of the given category of the given variable inside the totality of observations. A *P *value of 0.05 or less was considered statistically significant.

### Relationship between ozone concentration and temperature

For a more detailed analysis of the relationship between ozone concentration and temperature, we constructed a contingency table cross-classifying ozone concentration and temperature. Maximum values of ozone in each city were used.

Firstly, 1- and 8-hour ozone concentrations were cross-classified taking the value of 110 μg/m^3^/8h and the public information level of 180 μg/m^3^/h. In this way, the following situations were considered:

- Ozone concentrations below 110 μg/m^3^/8h corresponding to background pollution.

- Ozone concentrations above 110 μg/m^3^/8h but never reaching the 180 μg/m^3^/h level during the 8 hours. This situation is recognized as having potentially harmful effects on health, but the general public is non-informed in France.

- Ozone concentrations above 180 μg/m^3^/h, recognized in France as a pollution peak; the general public is informed.

For temperature, three different situations were considered:

- Temperatures below 26°C with no significant consequence on health.

- Temperatures above 26°C, demonstrated to be harmful to the health of patients with respiratory problems [16].

- Temperatures above 30°C, defined as a heat wave by meteorologists in France [16].

## Results

### Features of air pollution and weather between April 1 and September 30, 2003

Maximum 1- and 8-hour ozone concentrations were recorded in Chartres with 259 μg/m^3 ^and 240 μg/m^3 ^respectively (table 1). The highest ozone concentration and temperature and the lowest relative humidity in the three cities were recorded in August during the heat wave (table 1). All meteorological parameters were equivalent in the three cities (table 1). The winds came mainly from the north in Chartres, from the north east in Orléans and from the west in Tours.

### Characterization of ozone pollution data in relation to meteorological parameters from April 1 to September 30, 2003

**Figure 1:**
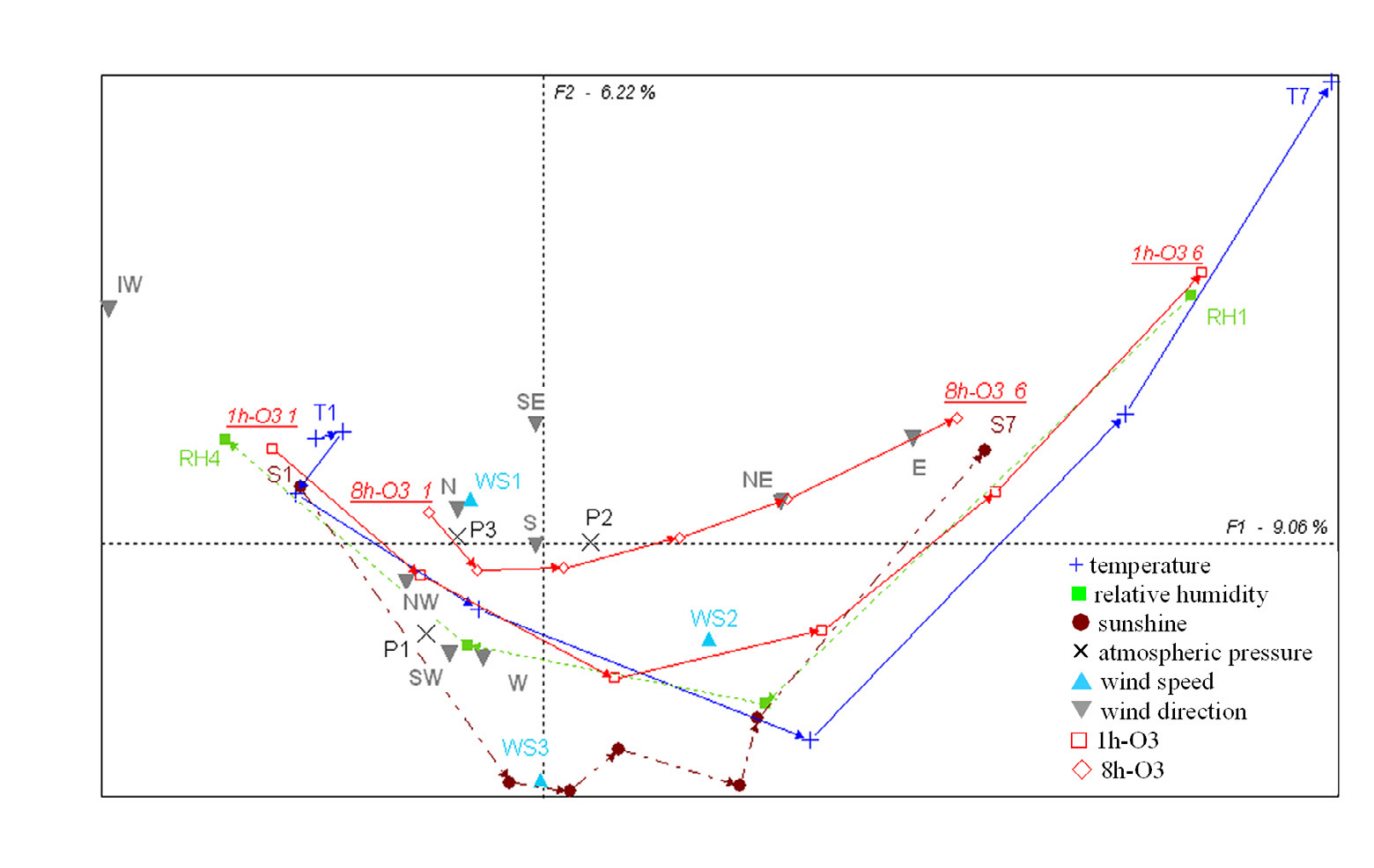
**Multiple Correspondence Analysis characterizing 1-and 8-hour ozone concentrations in relation to climate parameters, Chartres, April 1 to September 30, 2003**. This graph was plotted with 4166 statistical units (24 hours per day × 30 or 31 days per month × 6 months). Active variables are meteorological parameters: temperature (T, blue cross), relative humidity (RH, green bold square), sunshine (S, brown bold circle), atmospheric pressure (P, black cross), wind speed (WS, blue bold triangle) and wind direction (grey bold triangle). Illustrative variables are 1- (red square) and 8-hour ozone concentrations (red rhombus) (1h-O3 and 8h-O3 respectively). The proportion of the global variance expressed by F1 and F2 is indicated in italics. The indices beside each symbol refer the intensity from 1, the lowest values to the highest values.

Multivariate analyses characterizing 1- and 8-hour ozone concentrations in relation to meteorological parameters between April 1 and September 30, 2003 were carried out for Chartres, Orléans and Tours. The three cities had the same meteorological profile associated with highest 1- and 8-hour ozone concentrations. Therefore, only the results for Chartres are presented (figure 1).

The paths of 1-hour ozone concentration, temperature and relative humidity clearly overlap (figure 1). The lowest 1-hour ozone concentrations, lowest temperatures and highest relative humidity values are on the left of the F1 axis, while the highest 1-hour ozone concentrations, highest temperatures and lowest relative humidity values are on the right. Each category of 1-hour ozone concentration is in fact enclosed by one or more categories of temperature and relative humidity, indicating for example that ozone concentrations between 150 and 260 μg/m^3^/h are positively characterized by 0 – 40% relative humidity (p < 0.0001) and by temperatures between 26 and 41°C (p < 0.0001).

This indicates that ozone, temperature and relative humidity are strongly correlated: 1-hour ozone concentrations are positively correlated with temperature (r = 0.74) and negatively correlated with relative humidity (r = - 0.73).

Sunshine and 1-hour ozone concentrations are correlated (r = 0.46), with maximum minutes of sunshine per hour characterizing 1-hour ozone concentrations between 100 and 260 μg/m^3 ^(p < 0.0001).

Highest 1-hour ozone concentrations are characterized by winds from the north east (p < 0.0001) and east (p < 0.0001) but with no specific wind speed.

Finally, multivariate analysis shows that only the "normal atmospheric pressure" category characterizes 1-hour ozone concentrations between 80 and 260 μg/m^3 ^(p < 0.0001).

For 8-hour ozone concentrations, the corresponding path does not overlap temperature and relative humidity to the same extent as the 1-hour ozone concentration path (figure 1). However, highest 8-hour ozone concentrations also correspond to highest temperatures (p < 0.0001) and lowest relative humidity values (p < 0.0001). Like 1-hour ozone concentrations, highest 8-hour ozone concentrations are characterized by winds from the north east (p < 0.0001) and east (p = 0.002), by 50 to 60 minutes of sunshine per hour (p <0.0001) and by normal atmospheric pressure (p < 0.0001) with no particular wind speed.

### Characterization of ozone pollution measurements in relation to meteorological parameters in August 2003

A sub-analysis focused on August which was the hottest month in 2003. The highest temperatures were 39.4°C in Chartres and 39.5°C in Orléans and Tours.

**Figure 2:**
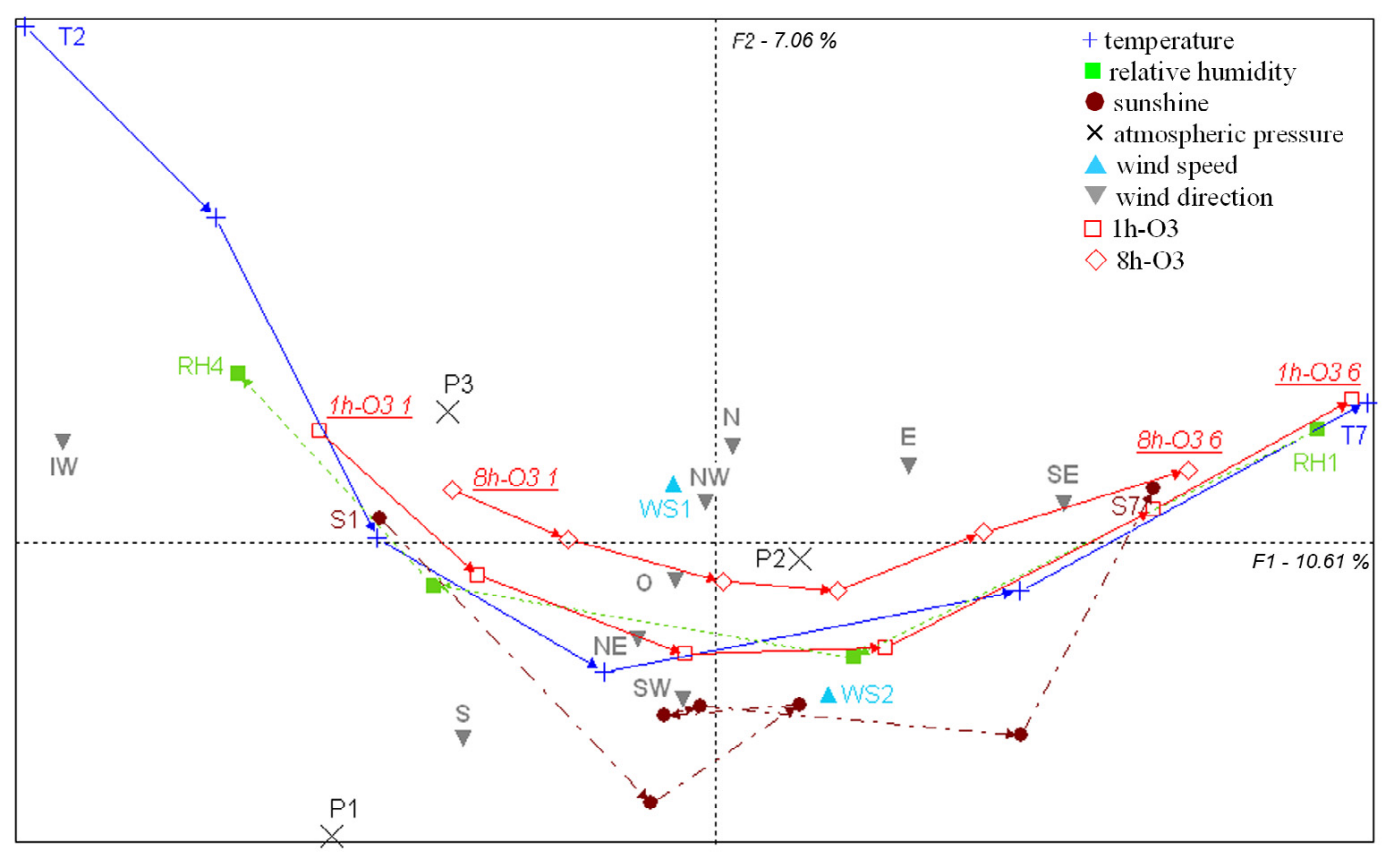
**Multiple Correspondence Analysis characterizing 1-and 8-hour ozone concentrations in relation to climate parameters, Tours, August 2003**. This graph was plotted with 744 statistical units (24 hours per day × 31 days × 1 month). Active variables are meteorological parameters: temperature (T, blue cross), relative humidity (RH, green bold square), sunshine (S, brown bold circle), atmospheric pressure (P, black cross), wind speed (WS, blue bold triangle) and wind direction (grey bold triangle). Illustrative variables are 1- (red square) and 8-hour ozone concentrations (red rhombus) (1h-O3 and 8h-O3 respectively). The proportion of the global variance expressed by F1 and F2 is indicated in italics. The indices beside each symbol refer the intensity from 1, the lowest values to the highest values.

Multivariate analyses were performed for the three cities with the same results. Thus, only the data for Tours are presented (figure 2).

Again, highest 1- and 8-hour ozone concentrations were characterized by highest temperatures (p < 0.0001) and lowest relative humidity values (p < 0.0001), by the maximum amount of sunshine per hour (p < 0.0001) and by normal atmospheric pressure (p < 0.0001). Neither highest 1-hour nor 8-hour ozone concentrations were associated with any specific wind speed. The highest 1-hour ozone concentrations were characterized by wind from the east (p = 0.004), north (p = 0.016) and south east (p = 0.035), whereas highest 8-hour ozone concentrations were characterized only by wind from the north (p = 0.009). In Orléans and Chartres (data not shown) highest 1-hour ozone concentrations were characterized by wind from the east (p = 0.007 and p < 0.0001 respectively) and north east (p < 0.0001 in Chartres only). Finally, 8-hour ozone concentrations were typically associated with wind from the north east (p < 0.0001 in Orléans and p = 0.001 in Chartres).

### Relationship between temperature and ozone concentration and consequences for interpreting the PAPRICA health study

Multivariate analyses showed a strong correlation between ozone concentration and temperature, which confirms that temperature must be considered as a confounding factor in the analysis of PAPRICA health data.

For a more detailed analysis of the relationship between ozone concentration and temperature, we cross-classified the category size of these two parameters (table 2), from which three situations were identified:

- According to table 2, ozone concentrations above 110 μg/m^3^/8h and temperatures below 26°C were recorded 432 times (indicated in bold type). This situation will allow the health impact of ozone pollution alone to be evaluated.

- Secondly, 164 temperatures over 26°C were associated with ozone concentrations below 110 μg/m^3^/8h (observations underlined). This situation will allow the health impact of high temperature alone to be evaluated.

- Finally, there are 265 observations corresponding to ozone concentrations above 110 μg/m^3^/8h associated with temperatures above 26°C (in italics). With this situation, characterized by both high ozone concentration and high temperature, it is not possible to determine whether the observed impact on health can be attributed to high ozone concentrations or to high temperatures.

The 5 observations of ozone concentrations above 180 μg/m^3^/h but below 110 μg/m^3^/8h correspond to events isolated in time (table 2).

## Discussion

The PAPRICA program seeks to assess the impact on the health of patients with chronic respiratory insufficiency of a prevention strategy based on information about ozone pollution. Ozone was chosen as a pollution indicator because it is the most damaging pollutant for air quality in the Centre Region of France. The first year of the PAPRICA study coincided with the summer 2003 heat wave. Thus, high temperatures have to be considered as a potential confounding factor in the analysis of our data and we had to find a method to avoid, or at least take into account, this confounding factor.

In most epidemiological studies on the health effects of ambient air pollution, pollutants and meteorological parameters to which the target population are exposed have to be included in the data analysis. The health impact of exposure to a specific pollutant is then assessed according to a methodology which typically uses environmental data in a regression model [2,9,17] or applies an exposure-response function derived from pooled analysis or published meta-analysis [18]. For studies focused on ozone effects, weather control is particularly important as high ozone days are generally quite hot. Schwartz [19] applied a case-crossover approach to study the impact of ozone on daily deaths in 14 US cities. The case-crossover approach consists in a case-control study whereby each person who had an event is matched with him- or herself on a nearby time period in which that individual did not have the event. This method controls for temperature by matching.

The PAPRICA study differs in that it involves an interventional aspect, our main focus being the effect on the health of patients with chronic respiratory insufficiency of a prevention strategy rather than the actual effect of ozone pollution itself. Thus, the design of this study involved two groups of patients, informed versus non-informed population. Therefore, traditional epidemiological methods are not suitable for analysing the PAPRICA data, which requires particular health data processing, discriminating between the effects of high ozone concentration and high temperature.

To study the relationship between ambient ozone concentration and temperature, we used multivariate analysis in order to identify associated climate and ozone pollution profiles. This confirmed that ozone production was associated with hot, dry weather, high ozone concentration being associated with high temperature, low relative humidity and prolonged sunshine. To study in more detail the relationship between temperature and ozone concentration, we cross-classified the category size of these two parameters in the following situations:

- Ozone concentrations above 110 μg/m^3^/8h but never reaching the 180 μg/m^3^/h level. At this level, French legislation does not require the general public to be informed, although it is recognized as having harmful effects on the health of people with respiratory disease.

- Ozone concentrations above 180 μg/m^3^/h which are considered as pollution peaks which are notified to the general public in France.

- Temperatures above 26°C corresponding to a discomfort level.

- Temperatures above 30°C, defined as a heat wave by meteorologists in France.

Even though high ozone concentrations were often associated with high temperatures, the reverse was not always true. The following three situations were identified: first, 432 pollution events (i.e. 10% of total observations) with temperatures below 26°C, from which the health impact of ozone pollution alone can be evaluated. Secondly, temperatures above 26°C not associated with ozone pollution (4% of total observations) from which the health impact of high temperatures alone can be evaluated. Thirdly, the dual effect of high ozone concentration and high temperature was observed 265 times (i.e. 6.4% of total observations), with ozone pollution associated with high temperature. In this situation, it is not possible to determine whether the observed impact is due to high ozone concentrations or high temperatures.

## Conclusion

This work assessed the relationship between ozone and climatic parameters (temperature, relative humidity, atmospheric pressure, sunshine, and wind speed and direction) in the specific case of the 2003 heat wave in the Centre Region, France. Despite the strong correlation between ozone and temperature, different kind of relationships between ozone concentrations and temperature, measured hourly, have been found. This will allow in further analyses to assess the respective impact of both ozone pollution and temperature on health during the 2003 heat wave.

## Competing interests

The author(s) declare that they have no competing interests.

## Authors' contributions

SL collected data, performed analysis, participated in the interpretation of data and drafted the manuscript. MDM participated in the conception and design of the study, provided statistical expertise and revised the manuscript. PD participated in the interpretation of data and helped to draft the manuscript. JB and NV participated in the design of the study and the statistical analysis. PC is an expert in air chemistry and revised critically the manuscript. VL conceived of the study, and participated in its design and coordination and revised the manuscript. All authors read and approved the final version.

## Pre-publication history

The pre-publication history for this paper can be accessed here:



## Figures and Tables

**Table 1:** Pollution and weather variables between April 1 and September 30, 2003

		Chartres	Orléans	Tours
1h-O_3 _(μg/m^3^)	Minimum	1	0	0
	Median	72	74.5	69
	Maximum	259	216	204
8h-O_3 _(μg/m^3^)	Minimum	3	3.5	0
	Median	72	74	68.5
	Maximum	240	200	194,5
Temperature (°C)	Minimum	-3.5	-3.8	-2.5
	Median	17.1	17	17.7
	Maximum	39.4	39.5	39.5
Relative humidity (%)	Minimum	16	17	16
	Median	69	71	69
	Maximum	100	100	99
Sunshine (minutes of sunshine per hour)*	Minimum	0	0	0
	Median	29	28	29.5
	Maximum	60	60	60
Wind speed (ms^-1^)	Minimum	0	0	0
	Median	3	3	3
	Maximum	9	13	13
Atmospheric pressure (hectopascal)	Minimum	999.5	1000	1000.1
	Median	1017.8	1018.1	1017.8
	Maximum	1032	1031.5	1031.2

*: medians were calculated for daylight hours, i.e. 6 am to 10 pm.

**Table 2:** Contingency table cross-classifying temperature and ozone concentration, Chartres, between April 1 and September 30, 2003

	Ozone concentrations			
	
	< 110 μg/m^3^/8h	< 110 μg/m^3^/8h	≥ 110 μg/m^3^/8h	≥ 110 μg/m^3^/8h
	< 180 μg/m^3^/h	≥ 180 μg/m^3^/h	< 180 μg/m^3^/h	≥ 180 μg/m^3^/h
T < 26°C	3300	0	**426**	**6**
26°C ≤ T ≤ 30°C	133	0	*117*	*18*
T > 30°C	31	5	*67*	*63*

Total size corresponds to 4166 observations. The discrepancy with the total size indicated in the "material and methods" section is due to missing data.

Figures in bold print correspond to the 432 situations with ozone concentrations higher than 110 μg/m^3^/8h and temperatures below 26°C. Underlined figures correspond to the 164 situations with temperatures above 26°C and ozone concentrations below 110 μg/m^3^/8h. Figures in italics correspond to the 265 situations with ozone concentrations above 110 μg/m^3^/8h and temperatures above 26°C.
